# Pharmacokinetics of Ganoderic Acids A and F after Oral Administration of Ling Zhi Preparation in Healthy Male Volunteers

**DOI:** 10.1155/2012/780892

**Published:** 2012-04-22

**Authors:** Supanimit Teekachunhatean, Sasinun Sadja, Chadarat Ampasavate, Natthakarn Chiranthanut, Noppamas Rojanasthien, Chaichan Sangdee

**Affiliations:** ^1^Department of Pharmacology, Faculty of Medicine, Chiang Mai University, Chiang Mai 50200, Thailand; ^2^Center of Thai Traditional and Complementary Medicine, Faculty of Medicine, Chiang Mai University, Chiang Mai 50200, Thailand; ^3^Department of Pharmaceutical Sciences, Faculty of Pharmacy, Chiang Mai University, Chiang Mai 50200, Thailand

## Abstract

The objectives of this paper were to evaluate the pharmacokinetics of ganoderic acids A and F after a single oral dose of the water extract of MG2-strain Ling Zhi (MG2FB-WE) and to assess the influence of food on the pharmacokinetics in 12 healthy male volunteers. This study was a single-dose, open-label, randomized, two-phase crossover study with at least 2 wk washout period. Each subject was randomly assigned to receive a single oral dose of 3,000 mg of MG2FB-WE in granular formulation dissolved in 200 mL of warm water, either under a fasting condition, or immediately after a standard breakfast (fed condition). Blood samples were collected immediately before and at specific time points until 8 h after MG2FB-WE administration. Plasma ganoderic acids A and F concentrations were determined by using liquid chromatography-mass spectrometry (LC-MS) technique. In conclusion, the pharmacokinetic profile of both ganoderic acids under a fasting condition was characterized by rapid absorption from the gastrointestinal tract (*T*_max_ at approximately 30 min) and a short elimination half-life (<40 min). Food significantly decreased *C*_max_ and delayed *T*_max_, but did not affect the extent of ganoderic acid A absorption. However, concomitant food intake markedly impeded both rate and extent of ganoderic acid F absorption.

## 1. Introduction

 The fruiting body of *Ganoderma lucidum *known as Ling Zhi in China, one of the most famous traditional Chinese medicinal mushroom, has been used extensively for longevity and health promotion in China and other Asian countries for thousands of years [1–3]. Although it is still not clear about Ling Zhi's mechanism on longevity and health promotion, Ling Zhi has been used for the prevention or treatment of various conditions and diseases such as anorexia, neurasthenia, insomnia, migraine, allergy, asthma, bronchitis, gastritis, hepatitis, nephritis, arthritis, lupus erythematosus, diabetes, hypertension, hypercholesterolemia, cardiovascular problems, and cancers [2, 3].

**Figure 1:**
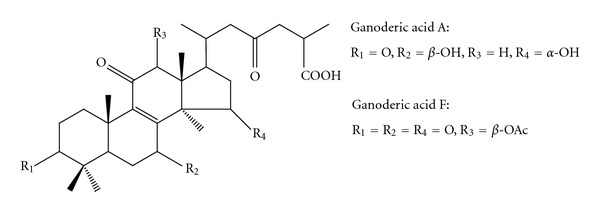
Structures of ganoderic acids A and F.

 Modern investigations have revealed that Ling Zhi contains a variety of phytochemical compounds. One of the potent biologically active compounds that has been shown to possess diverse and potentially significant pharmacological activities is the bitter triterpenes [2]. Since the first discovery of ganoderic acids A and B, more than 150 types of triterpenes have been isolated from various parts of Ling Zhi [2, 3], among which ganoderic acids A and F (Figure 1) have received considerable attention due to their conspicuous pharmacological properties for example, antihypertensive [4], antinociceptive [5], antioxidative [6], farnesyl protein transferase inhibitory [7], and hepatoprotective activities [8, 9], especially anticancer activity [10–13] which is the most attractive character of this medicinal mushroom. Ganoderic acid A has been reported to suppress growth and invasion of highly invasive human breast cancer cells via downregulation of expression of cyclin-dependent kinase 4 that regulates cell cycle G_1_ phase progression, and via suppression of secretion of urokinase-type plasminogen activator that implicates in tumor cell invasion and metastasis [12]. On the other hand, ganoderic acid F has exhibited antitumor and antimetastatic activities through inhibition of angiogenesis [10] and alteration of proteins involving cell proliferation and/or cell death, carcinogenesis, oxidative stress, calcium signaling, and endoplasmic reticulum stress [13]. 

Although several lines of scientific data from *in vitro* and *in vivo* studies supporting Ling Zhi's various pharmacological activities have been extensively documented, the pharmacokinetic study regarding its bioactive compounds in human have not yet been reported. Therefore, the purposes of this paper were to evaluate the pharmacokinetics of ganoderic acids A and F and the influence of food on their pharmacokinetics after an oral administration of the water extract of fruiting bodies of MG2-strain Ling Zhi (MG2FB-WE), produced by the Muang Ngai Special Agricultural Project under the patronage of Her Majesty Queen Sirikit, in healthy Thai male volunteers. This water extract of Ling Zhi in granular formulation is being under intensive investigation for its efficacy in the treatment of gynecologic and other cancers in the clinical trials conducted by the Faculty of Medicine, Chiang Mai University (CMU), Thailand.

## 2. Materials and Methods

### 2.1. Study Design

 The study was a single-dose, open-label, randomized, two-phase crossover study with at least a 2 wk washout period. This study was approved by the Human Research Ethics Committee of the Faculty of Medicine, CMU, and complied with the Declaration of Helsinki.

### 2.2. Subjects

 Twelve healthy Thai male subjects, aged between 18–40 y whose body mass index were within the normal range (18–25 kg/m^2^), were enrolled into this study. All subjects had to be considered healthy on the basis of their medical history and physical examination. The results of routine laboratory tests including complete blood count, liver function test, blood urea nitrogen, and creatinine had to be within normal limits. Subjects included in the study were given both verbal and written information regarding the nature and purpose of the study. Informed consent was voluntarily obtained from each subject prior to participation in the study. Exclusion criteria were subjects with known hypersensitivity to Ling Zhi, known medical history of neurological, pulmonary, kidney, liver, cardiovascular diseases, or malignancy, recent cigarette smoking within the previous 3 months, use of alcohol, substance abuse, any Ling Zhi preparation, as well as other medications (except acetaminophen) within the previous 1 month.

### 2.3. Dosage and Administration

 Eligible subjects were admitted to the Clinical Pharmacology Unit, Faculty of Medicine, CMU, at 6:30 AM after an overnight fast of at least 8 h. Each subject was randomized to receive a single oral dose of MG2FB-WE either under a fasting condition, or immediately after a Melander type standard breakfast (fed condition). The standard breakfast consists of 150 mL semiskimmed milk, 100 mL orange juice, 1 hard-boiled egg, 2 pieces of whole wheat bread, 5 g margarine, 20 g orange marmalade and 20 g hard cheese [14]. The 3,000 mg of MG2FB-WE in granular formulation containing 1,417.80 ± 40.74 *μ*g/g of ganoderic acid A and 224.15 ± 8.02 *μ*g/g of ganoderic acid F was dissolved in 200 mL of warm water before oral administration. All subjects were instructed to remain upright without intake of any food or beverage for 2 h after Ling Zhi administration. Water and lunch were served at 2 and 4 h after dosing, respectively. Serial blood samples were collected at different time points as described below. After blood sample collection at 8 h after dose, all subjects were discharged from the Clinical Pharmacology Unit. After a washout period of at least 2 wk, the subjects were crossed over to receive the same oral dose of Ling Zhi preparation after an alternative (fasting or fed) condition. The blood sample collection and other study conditions in the 2nd study period were as same as the previous study period. An identical meal and fluid were served on both study days. All subjects were instructed to avoid consumption of Ling Zhi or any Ling Zhi preparation throughout the study period.

### 2.4. Blood Sample Collection

 Serial blood sample collections (10 mL each) were obtained before oral administration of the Ling Zhi preparation, and at 5, 10, 15, 30, and 45 min, then at 1, 1.5, 2, 2.5, 3, 3.5, 4, 5, 6, and 8 h, respectively, after dosing for the determination of the plasma concentration of ganoderic acids A and F. The blood samples were obtained from the forearm by venipuncture through an indwelling intravenous catheter and collected into heparinized vacutainers. The blood collecting tubes were centrifuged at 1,040 g for 15 min at 4°C and the plasma was then separated and frozen at −20°C until analysis.

### 2.5. Determination of Ganoderic Acids A and F Concentrations

#### 2.5.1. Sample Preparation

**Figure 2:**
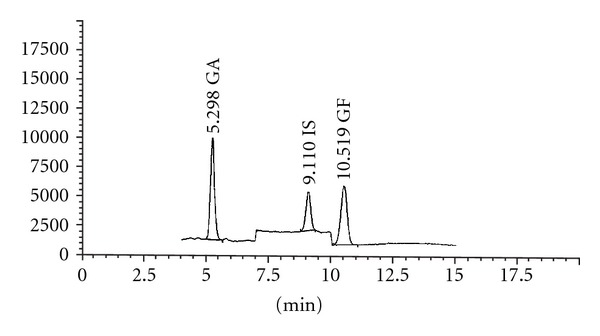
LC-MS chromatogram of plasma containing 18.00 ng/mL of ganoderic acids A (GA, retention time = 5.298 min) and F (GF, retention time = 10.519 min) as well as 2.50 ng/mL of IS (retention time = 9.110 min).

 The plasma sample extraction for the quantitative determination of ganoderic acids A and F was performed by using protein precipitation method. Concisely, 250 *μ*L of each plasma sample was spiked with 25 *μ*L of internal standard (IS, 2.50 ng/mL of cortisone 21-acetate), and subsequently deproteinated by mixing with 500 *μ*L of 1% acetic acid in 50% methanol/acetonitrile and then kept at room temperature for 20 min. The proteins in the plasma sample were separated by centrifuge at 18,620 g for 10 min at room temperature. Thereafter, an aliquot of the supernatant was removed and evaporated to dryness by the concentrator at 60°C for 1.5 h. The residues were then dissolved in 50 *μ*L of mobile phase and a 15 *μ*L of the sample was injected into the LC-MS system. LC-MS chromatogram of plasma containing ganoderic acids A and F and IS is presented in Figure 2. Plasma concentration of ganoderic acids A and F were determined by using a calibration curve of the peak area ratios of each ganoderic acid and IS, versus respective ganoderic acid concentrations with the use of linear regression analysis (correlation coefficient value ≥0.99). 

#### 2.5.2. LC-MS System and Conditions

All analyses were performed using an Agilent 1100 series LC system coupled with MSD single quadrupole mass spectrometer (Agilent LC/MSD API-Electrospray) system. The sample were separated using a Zorbax SB-C_18_ analytical column (4.6 × 150 mm, 5 *μ*m) from Agilent technologies in a 20 min runtime. Solvent A consisted of 10 mM of ammonium formate (pH 4.00), whereas solvent B was acetonitrile. The mobile phase was delivered in a constant ratio of solvent A : B (40 : 60, v/v) at flow rate of 1.0 mL/min. The MS was equipped with an electrospray ionization interface and operated in positive ion mode in mass-to-charge radios of 499.40 and 555.30 m/z for ganoderic acid A, 571.30 and 572.30 m/z for ganoderic acid F and 403.20 and 441.20 m/z for IS. The gas temperature was 350°C, drying gas 13 L/min, and nebulizer pressure 50 psi.

### 2.6. Assay Validation

 The assay validation was performed following the US Food and Drug Administration guidance for bioanalytical method validation [15]. The LLOQ value of both ganoderic acids A and F under the LC-MS condition used in this study was 0.50 ng/mL. The percentages of coefficient of variation (% CV) at LLOQ concentration of ganoderic acids A and F were 5.92% and 15.90%, respectively, whereas the accuracy at this concentration were 113.50% and 114.95%, respectively. The mean % CV of intraday precision of ganoderic acids A and F were 3.48% and 3.62%, respectively, whereas, those of interday of ganoderic acids A and F were 3.64% and 3.17%, respectively. Likewise, the mean accuracy of intraday assay validation of ganoderic acid A and F were 105.16% and 101.38%, respectively, whereas those of interday of ganoderic acids A and F were 103.01% and 99.35%, respectively. The mean recovery of ganoderic acids A and F including IS were 73.51%, 89.52%, and 72.66%, respectively.

### 2.7. Data Analysis and Statistical Methods

#### 2.7.1. Pharmacokinetic Parameters

 The maximum plasma concentration (*C*_max_, ng/mL) and time to reach maximum concentration (*T*_max_, h) of ganoderic acids A and F were evaluated directly by visual inspection of each subject's plasma concentration-time profile. The area under the concentration-time curve from administration to 8 h and to infinity (AUC_0–8_  and AUC_0–*∞*_, ng·h/mL) as well as elimination half-life (*t*_1/2_, h), were determined by non-compartmental analysis. The slope of the terminal log-linear portion of the concentration-time profile was determined by least-squares regression analysis and used as the elimination rate constant (*K*_*e*_). The elimination *t*_1/2_ was calculated from the ratio of 0.693/*K*_*e*_. The AUC from time zero to the last quantifiable point (AUC_0–8_) was calculated by using the trapezoidal rule and the extrapolated AUC from time *t* to infinity (AUC_*t*–*∞*_) was determined as *C_t_*/*K_e_*. Total AUC was the sum of AUC_0–8_ + AUC_8–*∞*_. The calculation was performed by using the Topfit software version 2.0 for personal computer. 

#### 2.7.2. Statistical Analysis

 The pharmacokinetic parameters were presented as mean ± standard deviation (SD). The differences in the mean values of *C*_max_, *T*_max_, *t*_1/2_, AUC_0–8_, and AUC_0–*∞*_ between fasting and fed conditions were analyzed by using paired Student's *t*-test and were considered statistically significant if *P* < 0.05.

## 3. Results

 The demographic characteristics and means of clinical laboratory data of 12 subjects enrolled in the study are shown in Table 1. All subjects completed the study protocol. On the basis of medical history, physical examination and laboratory investigation, none of the subjects showed any evidence of neurological, pulmonary, kidney, liver, or cardiovascular diseases.

**Figure 3:**
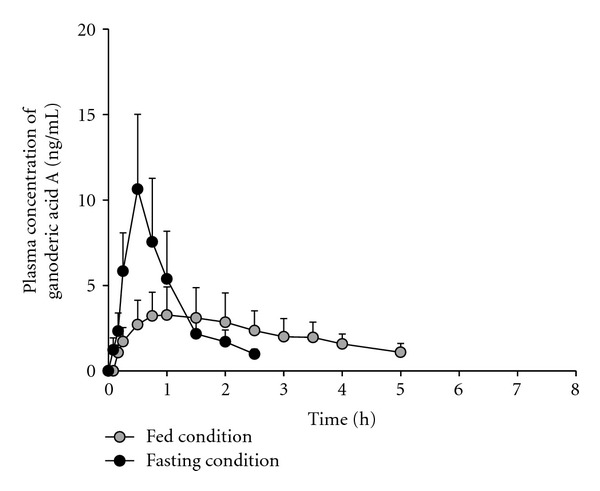
Mean plasma ganoderic acid A concentration-time curves after a single oral dose of MG2FB-WE under a fasting or fed condition.

**Figure 4:**
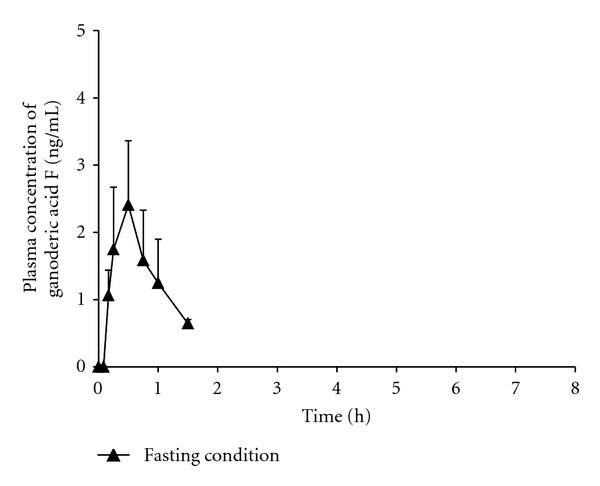
Mean plasma ganoderic acid F concentration-time curve after a single oral dose of MG2FB-WE under a fasting condition (mean plasma ganoderic acid F concentration-time curve under a fed condition is not shown due to insufficient data for calculation).

 The mean plasma concentration-time curves of ganoderic acid A at various sampling times from 12 subjects after a single oral administration of 3,000 mg of MG2FB-WE under a fasting or fed condition are presented in Figure 3. That of ganoderic acid F under a fasting condition is presented in Figure 4. However, the mean plasma ganoderic acid F concentration-time curve under a fed condition is not shown due to insufficient data for calculation since plasma ganoderic acid F concentrations that are higher than the LLOQ were detected only in 2 out of 12 subjects. In one subject, the concentrations of 0.59 and 0.50 ng/mL were found at 2.50 h and 3.00 h after MG2FB-WE administration, respectively, whereas the concentration of 0.56 ng/mL was found at 3.50 h after dosing in another subject.

 The pharmacokinetic parameters of ganoderic acids A and F (*C*_max_, *T*_max_, *t*_1/2_, AUC_0–8_, and AUC_0–*∞*_) following a single oral administration of 3,000 mg of MG2FB-WE under a fasting or fed condition are presented in Table 2. Under a fasting condition, both ganoderic acids reached their *T*_max_ at approximately 30 min. Ganoderic acids A and F had a very short elimination *t*_1/2 _ of 37.20 min and 28.80 min, respectively. Food significantly decreased *C*_max_ as well as delayed *T*_max_ and *t*_1/2_, but did not affect the extent (AUC_0–8_ and AUC_0–*∞*_) of ganoderic acid A. However, since the plasma ganoderic acid F concentrations at any time points under a fed condition were below the LLOQ in most of the enrolled subjects, the pharmacokinetic parameters such as *C*_max_, *T*_max_, *t*_1/2_, AUC_0–8__  _  including AUC_0–*∞*_ were not be determined and therefore were not be statistically compared with those under a fasting condition.

## 4. Discussion

 This is the first report on the pharmacokinetic study of ganoderic acids A and F after a single oral administration of MG2FB-WE in healthy Thai male volunteers. Since the study design of this pharmacokinetic study was similar to that of bioequivalence testing, the minimum number of 12 subjects were enrolled in the study according to the guideline on investigation of bioequivalence [16]. Additionally, a two-phase crossover study was conducted in order to minimize subject variability between fasting and fed conditions.

 The MG2FB-WE used in our pharmacokinetic study is currently under intensive investigation at the Faculty of Medicine, CMU, for its efficacy in the treatment of advanced gynecologic and other advanced-stage cancers using a dosage of 3,000 mg twice daily (6,000 mg/day) for 3 months. This dosage was selected in accordance to the study previously reported by Gao et al. [17] exhibiting that the oral administration of 5,400 mg/day of Ling Zhi extract for 12 wk significantly enhances the immune responses in patients with advanced-stage cancers. Indeed, MG2FB-WE used in the ongoing clinical trials was prepared as granular formulation dissolved in 200 mL of warm water before oral administration. This formulation has proved to be easier and more acceptable for the cancer patients to consume than other dosage forms, such as a single dose of 6 capsules (500 mg/capsule) each time. Therefore, a single dosage of 3,000 mg of MG2FB-WE in granular formulation was investigated in the present study based on the dosage and formulation used in the ongoing clinical trials mentioned above. In addition, the granular formulation was considered to be superior to other formulations (capsules and tablets) in this pharmacokinetic study because the granules are readily dissolved and absorbed without the necessity to evaluate for its dissolution and disintegration profiles, which are the major confounding factors during an absorptive phase.

 The measurement of plasma ganoderic acids A and F was performed by using the LC-MS method due to its rapid (runtime of 20 min), high specificity and sensitivity (LLOQ of 0.50 ng/mL) in comparison to longer runtime (runtime of 60 min) and lower sensitivity (LLOQ of 2.50 *μ*g/mL) by HPLC-UV (252 nm) technique in our preliminary experiments. The validation of LC-MS assay demonstrated validity in precision, accuracy as well as recovery following the US FDA guidance, thus showing the suitability of this method for analysis of ganoderic acids A and F in plasma samples.

 According to plasma concentration-time curves under a fasting condition, ganoderic acids A and F could be detected in the plasma as early as 5–10 min after an oral administration and reached their *T*_max_ at approximately 30 min. Both ganoderic acids A and F had a very short elimination *t*_1/2_ of 37.20 min and 28.80 min, respectively. These findings are in agreement with the previously reported pharmacokinetic parameters in animals that revealed rapid absorption and elimination of *G. lucidum* triterpenes as evidenced by a *T*_max_ value range from 18–110 min and elimination *t*_1/2_ of 35–143 min [18–20]. AUC of ganoderic acids A and F were low in spite of large dose of Ling Zhi preparation containing relatively high contents of ganoderic acids A (4253.40 *μ*g) and F (642.75 *μ*g) was administered. This data suggests low oral bioavailability of ganoderic acids A and F, which was consistent to the bioavailability of approximately 10% of ganoderic acid A reported in previous studies [18]. Since the rate of drug absorption is almost always directly proportional to the extent of absorption, we therefore postulated that the relatively low oral bioavailability of ganoderic acids A and F could probably not result from the poor absorption from gastrointestinal tract because their absorption appeared to be very rapid. However, this poor oral bioavailability might be due to their extensive hepatic first-pass metabolism coupled with partial conversion of some triterpenes to their metabolites by intestinal bacteria as reported in rat feces, but not in plasma and urine [20]. Further studies should be investigated to identify the exact mechanisms involving in this low oral bioavailability.

 It is well known that food may positively or negatively affect the rate and/or extent of the bioavailability of various drugs [21–23]. This study revealed that food caused a significant decrease in *C*_max_ and rate of absorption (*T*_max_) of ganoderic acid A, but not the extent (AUC) of absorption. Since it is established that most drugs are ordinarily absorbed from the small intestine and delayed gastric emptying will delay absorption of those drugs that are absorbed predominantly from the small intestine [21, 23], we postulated that food affected the rate but not the extent of ganoderic acid A absorption through slowing of gastric emptying. Indeed, many dietary factors, such as solid food, high-fat content, and high osmolarity, have been found to delay gastric emptying [21–23]. Nonetheless, concomitant food administration also significantly prolonged the *t*_1/2 _ of ganoderic acid A. This finding presumably resulted from the delayed ganoderic acid A absorption due to prolonged gastric emptying by food, yielding sustained plasma levels and distorted the terminal *t*_1/2_ under a fed condition.

 The absorption of ganoderic acid F was probably affected by food in the same manner as that of ganoderic acid A. Since the concentrations of ganoderic acid F were already low under a fasting condition, the effect of food would then impede the absorption to the point that its plasma concentrations were lower than the LLOQ. Its pharmacokinetic parameters, likewise, could not be assessed. Owing to the fact that food intake generally impairs the rate and/or extent of ganoderic acids and perhaps other triterpenes, we recommend that Ling Zhi preparations should be taken on an empty stomach whenever possible.

 Several *in vitro* studies have demonstrated that cytotoxicity against various human cancer cell lines expressed as IC_50_ values are in the range of 9.47–26.50 *μ*M (approximately 4,900–13,700 ng/mL) for ganoderic acid A [11] and 9.62–19.50 *μ*M (approximately 5,500–11,000 ng/mL) for ganoderic acid F [11, 13]. These targeted concentrations are much higher than the mean *C*_max_ of 10.99 ± 4.02 ng/mL for ganoderic acid A and 2.57 ± 0.91 ng/mL for ganoderic acid F found in this study. Therefore, it is unlikely to achieve cytotoxic effects *in vivo* although a relatively high-dose or multiple-dosage regimen of Ling Zhi extract is used. However, Ling Zhi is well documented to contain over 150 types of triterpenes [2, 3], and many of them have been demonstrated to possess direct anticancer activity through different mechanisms of action for example, induction of cell cycle arrest and apoptosis [24, 25], inhibition of proliferation, migration, invasion, metastasis, and angiogenesis of carcinoma cell lines [10, 12, 13, 26]. Therefore, *in vivo* anti-cancer activity might be exerted via synergistic effects among these triterpenes and with other biologically active compounds such as immunomodulatory protein, Ling Zhi-8 [27, 28]. Additionally, polysaccharide fractions might also play some additional benefits through activation of an immune response against cancer [29, 30].

 The major limitation of present study was the limited ability of the LC-MS technique to measure very low levels of ganoderic acid F in plasma samples, especially under a fed condition, because its plasma concentrations at any time points were lower than the LLOQ value of an analytical method, being unable to establish individual plasma concentration-time data and hence calculation for pharmacokinetic parameters. Therefore, a more sensitive analytical method (such as LC-MS/MS) or study using a Ling Zhi preparations containing high content of ganoderic acid F are suggested for the determination of human plasma ganoderic acid F and other triterpenes concentrations in future studies.

## 5. Conclusion

The pharmacokinetic profile of both ganoderic acids under a fasting condition was characterized by rapid absorption from the gastrointestinal tract (*T*_max_ at approximately 30 min) and a short elimination half-life (<40 min). Food significantly decreased *C*_max_ and delayed *T*_max_, but did not affect the extent (AUC_0–8_  and AUC_0–*∞*_) of ganoderic acid A absorption. However, concomitant food intake markedly impeded both rate and extent of ganoderic acid F absorption.

## Figures and Tables

**Table 1:** The demographic characteristics and clinical laboratory data of 12 subjects enrolled in the study.

Parameters	Mean ± SD	Range	Normal values
Age (y)	26.83 ± 4.86	20–33	
Weight (kg)	58.38 ± 7.29	48.5–74.0	
Height (m)	1.69 ± 0.06	1.60–1.78	
BMI (kg/m^2^)	20.51 ± 1.97	18.25–23.36	18–25
Laboratory data			
Hemoglobin (g/L)	145.50 ± 6.49	130–157	130–180
Hematocrit (L/L)	0.45 ± 0.02	0.43–0.48	0.40–0.54
WBC (×10^9^/L)	7.93 ± 1.83	5.6–11.0	4.4–11.0
Platelets on smear	Adequate	—	Adequate
BUN (mg/dL)	13.17 ± 1.95	10–17	8.4–21
Creatinine (mg/dL)	1.08 ± 0.11	0.9–1.3	0.8–1.3
SGOT (U/L)	21.58 ± 4.54	16–30	0–37
SGPT (U/L)	23.50 ± 9.13	13–39	0–41
ALP (U/L)	47.50 ± 11.02	30–64	53–128
Total bilirubin (mg/dL)	0.42 ± 0.23	0.2–0.9	0.1–1.2

BMI: body mass index; WBC: white blood cell; BUN: blood urea nitrogen; SGOT: serum glutamic oxaloacetic transaminase; SGPT: serum glutamic pyruvic transaminase; ALP: alkaline phosphatase.

**Table 2:** Pharmacokinetic parameters of ganoderic acids A and F after a single oral administration of MG2FB-WE under a fasting or fed condition.^†^

Parameters	Ganoderic acid A		Ganoderic acid F	
	Fasting condition	Fed condition	Fasting condition	Fed condition
*C*_*max*_ (ng/mL)	10.99 ± 4.02**	3.84 ± 1.56	2.57 ± 0.91	ND
*T*_*max*_ (h)	0.54 ± 0.18*	1.67 ± 0.88	0.52 ± 0.13	ND
*t*_1/2_ (h)	0.62 ± 0.17*	1.34 ± 0.65	0.48 ± 0.22	ND
AUC_0–8_ (ng·h/mL)	9.58 ± 4.08	8.75 ± 5.32	1.81 ± 0.76	ND
AUC_0–*∞*_ (ng·h/mL)	10.53 ± 4.32	11.02 ± 5.54	2.42 ± 0.93	ND

^†^ Data represents mean ± SD.

*P < 0.01, **P < 0.001 denote statistically significant as compared to a fed condition according to paired Student's *t*-test.

ND: cannot be determined because the plasma ganoderic acid F concentrations at any time points were below the LLOQ in most of the enrolled subjects.

## Abbreviations

AUC_0–*∞*_: Area under the concentration-time curve from administration to 8 h
AUC_0–*∞*_: Area under the concentration-time curve from administration and extrapolation to infinity
*C*_max⁡_: Maximum plasma concentration
*C_t_*: Concentration at time *t *
*K_e_*: Elimination rate constant
*t*_1/2_: Half-life
*T*_ max_: Time to reach maximum concentration.
